# Microclimate Characterization of a Low-Tech Greenhouse During a Tomato Crop (*Solanum lycopersicum* L.) Production Cycle in Chaltura, Imbabura

**DOI:** 10.3390/plants14233702

**Published:** 2025-12-04

**Authors:** Luis Marcelo Albuja-Illescas, Miguel Gómez-Cabezas, Gabriel Jácome-Aguirre, Juan Pablo Aragón-Suárez, Rafael Jiménez-Lao, Araceli Peña-Fernández, María Teresa Lao

**Affiliations:** 1 Agrobiodiversity and Food Security Research Group—GIASSA, Agricultural and Environmental Science Faculty, Universidad Técnica del Norte, Av. 17 de Julio 5-21 y Gral. José María Córdova, Ibarra EC100105, Ecuador; magomez@utn.edu.ec (M.G.-C.); jparagon@utn.edu.ec (J.P.A.-S.); 2 Geosciences and Environmental Laboratory (GEOMA), Faculty of Agricultural and Environmental Engineering, Universidad Técnica del Norte, Av. 17 de Julio 5-21 y Gral. José María Córdova, Ibarra EC100150, Ecuador; gajacomea@utn.edu.ec; 3 Department of Engineering, University of Almería, 04120 Almería, Spain; rjlao717@gmail.com; 4 CIAIMBITAL Research Centre, Engineering Department, University of Almería, 04120 Almería, Spain; apfernan@ual.es; 5 Agronomy Department, Research Center for Mediterranean Intensive Agrosystems and Agrifood Biotechnology CIAMBITAL, Agrifood Campus of International Excellence ceiA3, University of Almería, 04120 Almería, Spain; mtlao@ual.es

**Keywords:** greenhouse microclimate, vapor pressure deficit (VPD), microclimatic variability, protected agriculture, environmental monitoring, Andean horticulture

## Abstract

Greenhouse agriculture is experiencing global expansion; however, in Andean countries such as Ecuador, its development is constrained by low-tech infrastructure, limited automation, and insufficient environmental monitoring, all of which negatively affect productivity and fruit quality. This study characterized the microclimate of a low-tech greenhouse in Chaltura, Imbabura Province, during a complete production cycle of tomato crop (*Solanum lycopersicum* L.). Microclimatic conditions were analyzed during three phenological stages (vegetative, reproductive, and harvest). Temperature and relative humidity were recorded at 5 min intervals using sensors placed in the greenhouse quadrants, while an external weather station provided daily outdoor climate data. Statistical analyses were performed in R software (version 4.4.x). The results revealed marked internal microclimatic heterogeneity and showed that the crop remained outside the optimal ranges of temperature, relative humidity, and vapor pressure deficit (VPD) for over 50% of the time across all phenological stages and greenhouse quadrants. These findings underscore the urgent need for cost-effective climate-control strategies adapted to local conditions and provide a scientific basis for future research aimed at improving climatic and productive efficiency, as well as the resilience and sustainability of protected agriculture in Andean regions.

## 1. Introduction

Protected horticulture in the Andean province of Imbabura, Ecuador, is predominantly practiced in low-tech greenhouses. These structures, often built using artisanal methods, typically consist of metal or wooden frames as structural elements and are covered with polyethylene (PE) film, equipped with manual ventilation and drip irrigation systems [1]. Compared with open-field cultivation, this production system offers more controlled conditions that enhance crop yield and quality, including improved pest and disease management [2,3]. In the Andean region of Imbabura, low-tech greenhouses are mainly used for tomato (*Solanum lycopersicum* L.), which accounts for approximately 76.9% of the cultivated area. Other crops include pepper (*Capsicum annuum* L.) at 13.7%, cucumber (*Cucumis sativus* L.) at 5.6%, and, more recently, minor crops such as blueberry, strawberry, blackberry, grapes, and celery [1].

The primary purpose of protective structures is to adapt local climatic conditions to the specific requirements of the crop. The level of technological complexity depends on the degree of environmental modification needed.

The benefits of low-tech protected horticulture have been widely documented in different regions. Naturally ventilated greenhouses have been shown to increase productivity by improving plant physiological status through the optimization of environmental conditions [2,3,4]. Such systems also extend the production season, allow off-season supply and improve profitability for smallholder farmers, and enhance product quality [4,5]. Moreover, greenhouses are recognized as an important strategy to strengthen food and nutritional security in areas vulnerable to climate change [6].

However, the efficiency of these systems depends on the adequate control of critical microclimatic variables such as temperature, relative humidity, solar radiation, and wind speed, whose interactions determine the internal environment [7,8]. Low-tech greenhouses are not suitable for all climatic contexts and often require complementary strategies to maximize their effectiveness [2]. Their profitability also depends on balancing operational costs with yield increases and market prices, while considering environmental concerns such as groundwater contamination and soil degradation caused by inadequate management [5].

Covered environments are inherently heterogeneous, showing significant spatial and temporal variations in microclimatic conditions. This variability can adversely affect plant physiology, yield, and fruit quality [9,10,11,12]. In Colombia and other Latin American countries, studies have reported that artisanal greenhouses often exhibit uneven internal microclimates due to factors such as geometry, orientation, cover type, and management practices [11,13,14,15]. Variations in temperature and vapor pressure deficit (VPD) can lead to irregular plant and fruit development or induce physiological stress under extreme conditions, particularly during summer [12,16].

In Ecuador, greenhouses generally lack climate monitoring systems, and control decisions are based on empirical knowledge [1]. This absence of monitoring can lead to inefficient microclimate management, reinforcing the recommendation to incorporate sensor networks to support decision-making [12,16,17]. Proper environmental control can not only enhance productivity but also optimize resource use and reduce energy costs, which may represent up to 40% of total production expenses in other latitudes [18,19].

Numerous studies have demonstrated the effectiveness of predictive models and emerging technologies such as digital sensors, inference systems, and monitoring networks for greenhouse climate management [20,21,22,23]. However, in Ecuador, there is a lack of studies that document the spatial and temporal patterns of microclimates in low-tech systems. This knowledge gap limits the development of locally adapted management strategies and predictive models [11,13,24].

Low-tech greenhouses face major thermal challenges, including daytime overheating, nighttime cooling, and thermal inversion, particularly in structures without climate-control technology [25,26]. Consequently, microclimatic characterization is essential for establishing design criteria, improving production efficiency, and advancing sustainable development in protected horticulture [27,28].

In this context, the objective of this study was to characterize the microclimate of a low-tech greenhouse located in Chaltura (Imbabura) during a full production cycle of tomato (*Solanum lycopersicum* L.), the predominant crop in the region, as a basis for improving production efficiency under these conditions. To achieve this, the study also incorporated machine learning tools, specifically decision tree models, to categorize microclimatic conditions. This approach is particularly relevant given the limited number of agricultural studies that apply such methods, providing an innovative framework for analyzing complex microclimatic interactions in low-tech greenhouse systems.

## 2. Materials and Methods

### 2.1. Study Area Description

**Figure 1:**
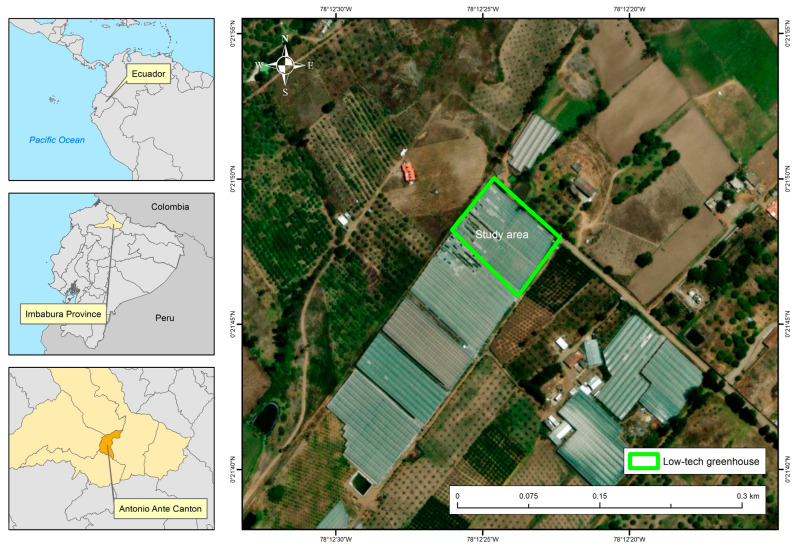
Geographical location of Greenhouse Block 2 at El Diamante Farm.

The study was conducted at the “El Diamante” farm, located in San José de Chaltura, Antonio Ante Canton, Imbabura Province, Ecuador, with coordinates 0°21′47.6″ N and 78°12′24.1″ W, at an elevation of 2370 m a.s.l. According to the Köppen–Geiger climate classification [29], the prevailing climate in this location corresponds to a Csb type. The average annual temperature is approximately 15.5 °C, with a total annual precipitation of 714 L m^−2^ [30]. The analysis was carried out in a low-tech greenhouse, designated as Block 2, which is part of the farm’s production complex. The geographical location of the study area is shown in Figure 1.

#### Greenhouse Design Characteristics

The surface of greenhouse is 6468 m^2^ and presents an asymmetrical flat-roof design, with a concrete foundation and wooden columns (*Eucalyptus globulus*) measuring 42 cm in diameter. It is equipped with a ridge vent and 80% white shade mesh on the lateral, front, and rear ventilation openings. The covering material consisted of long-lasting polyethylene (PE) film, with the following characteristics: thickness of 150 µm, photosynthetically active radiation (PAR) light transmission of 88%, diffuse PAR transmission of 56%, and thermicity of 75%.

**Figure 2:**
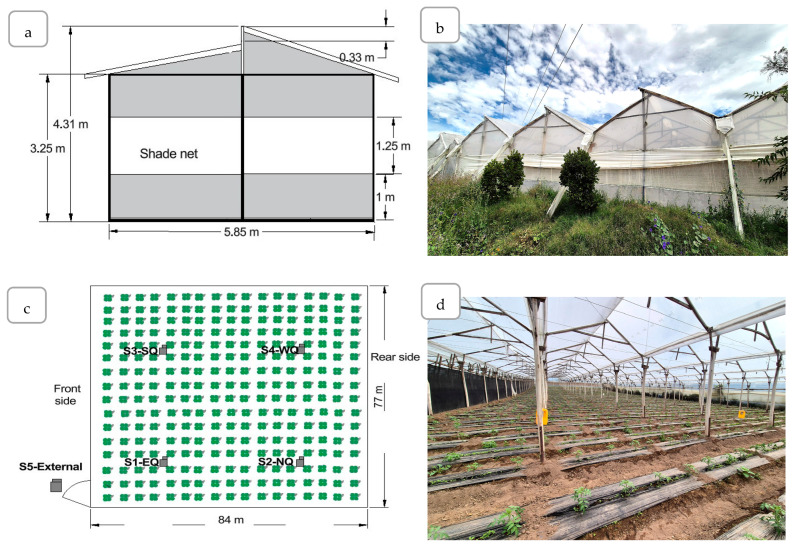
Structural layout of the greenhouse and spatial distribution of climatic sensors. (**a**) Scheme of frontal perspective. (**b**) Frontal exterior view. (**c**) Position of the data loggers. (**d**) Interior view. Note: S1-EQ = Eastern Quadrant; S2-NQ = Northern Quadrant; S3-SQ = Southern Quadrant; S4-WQ = Western Quadrant.

The greenhouse structure had a central front height of 4.31 m and a rear height of 4.72 m, resulting in a slope of 0.48%. The total ventilation area represented 10.46% of the surface area. On its left side, the structure was separated by less than 2 m from an adjacent greenhouse of identical dimensions, which could partially restrict lateral air exchange and limit the efficiency of natural ventilation. The longitudinal axis of the greenhouse was oriented at 317° toward the northwest (NW). The irrigation system used was drip irrigation (Figure 2).

### 2.2. Equipment for Climate Data Recording

#### 2.2.1. Internal Temperature and Relative Humidity Sensors

Four CS300-TH sensors (Milesight, Xiamen, China) were installed to monitor the internal microclimate. These sensors, designed for environmental monitoring using LoRaWAN^®^ technology, incorporate a high-precision Sensirion chip and are housed in an IP67-rated casing, resistant to both water and UV radiation. The technical specifications of the sensors are provided in Appendix A.

The sensors were positioned equidistantly above the crop canopy in a uniformly distributed peripheral arrangement, following the recommendations of [7,31,32].

#### 2.2.2. Weather Station for External Climate Records

External climate monitoring was conducted with a Davis Instruments Vantage Vue^®^ weather station (Davis Instruments, Hayward, CA, USA), connected to the WeatherLink network. This station recorded key environmental variables, including temperature, relative humidity, atmospheric pressure, wind speed and direction, and precipitation. Daily data were collected from March to September 2024. The technical specifications of the Davis station are presented in Appendix B.

Due to the absence of local radiation sensors at the greenhouse site, solar radiation data were obtained from the nearest meteorological station, located 4 km away and managed by a private producer. The station is situated at a comparable elevation and within the same agroecological zone, ensuring adequate spatial representativeness for this study.

### 2.3. Microclimatic Requirements of Tomato (Solanum lycopersicum L.)

The optimal and suboptimal ranges for temperature (T), relative humidity (RH), and vapor pressure deficit (VPD) for tomato were considered according to the crop’s phenological stages and periods of the day (daytime and nighttime). These thresholds are summarized in Table 1 and were adapted from [33].

### 2.4. Agronomic Parameters

The crop was established on raised beds covered with black plastic mulch. The planting density was 1.14 plants per square meter, trained to four stems. The cultivar used was Pietro F1 grafted in an Empower^®^ rootstock. Transplanting was performed on 14 March 2024. The cultivar Pietro F1 was selected for this study due to its widespread use among greenhouse tomato producers in the Imbabura province. Tomato is the principal crop grown under protected conditions in the region, and Pietro F1 is recognized for its adaptability to local agroclimatic conditions and market preference.

The recorded phenological stages included vegetative growth, reproductive development, and harvest, with a total crop cycle of 190 days, as shown in Table 2.

The yield was classified as a function of quality categories according to the local market: first, second, third and fourth.

The crop was irrigated using a drip irrigation system, which delivered water directly to the root zone through emitters installed along the planting lines. Fertilization was carried out via fertigation, using the same drip system to apply water-soluble nutrients throughout the crop cycle. This method allowed for efficient nutrient delivery and is commonly used in low-tech greenhouse systems in the region. The fertilization regime followed the grower’s standard protocol, adjusted according to phenological stage and visual crop assessment.

### 2.5. Data Processing and Analysis

#### 2.5.1. Internal Sensors

Temperature and relative humidity were recorded every 5 min from 14 March to 19 September 2024, generating approximately 200,000 data points across all internal sensors. Data processing and visualization were carried out in R software (version 4.4.x) [34]. Missing data patterns were visualized with the vis_miss function of the visdat package [35]. Although the proportion of missing data was low, it varied among sensors: S1-EQ (0.9%, ~3% for temperature and relative humidity), S2-NQ (0.7%, ~2%), S3-SQ (2.9%, ~10%), and S4-WQ (2.3%, ~8%).

Imputation was performed independently for each dataset using the missForest algorithm, a nonparametric random forest approach for mixed-type data [36]. All available predictors (phenological stage, day/night indicator, date components, and climate variables) were included to leverage multivariate relationships without imposing parametric assumptions. Default hyperparameters were applied (ntree = 100, automatic mtry, maximum 10 iterations) with a fixed random seed for reproducibility. Convergence was evaluated using the out-of-bag (OOB) normalized root mean squared error (NRMSE), with imputation stopped when no further improvement was observed.

The low initial proportion of missing data combined with strong inter-variable dependencies allowed accurate and plausible imputations, ensuring data integrity for subsequent analyses. Based on these records, descriptive statistics and VPD were calculated and disaggregated by phenological stage.

Following the methodology of Noh and Lee (2022) [37], VPD was computed from T and RH according to Equations (1)–(3):

(1) $$SVP\;=\;0.6108\;\times \;2.7183^{\left[(17.27\;\times \;T)/(T+237.3)\right]}$$

(2) AVP=SVP×(RH100)

(3) VPD=SVP−AVP

where SVP is the saturation vapor pressure, T the air temperature (°C), AVP the actual vapor pressure, RH the relative humidity (%), and VPD the vapor pressure deficit (kPa).

To test spatial variability, one-way analysis of variance (ANOVA), was applied to temperature, relative humidity, and vapor pressure deficit across sensors. Statistically significant differences (*p* < 0.05) were considered evidence of spatial heterogeneity. Boxplots were generated for each variable to visually represent data distribution and highlight potential outliers, aiding in the interpretation of greenhouse microclimatic variability.

#### 2.5.2. Weather Station

External climate data were collected daily throughout the crop cycle and descriptive statistics were computed using R software (version 4.4.x).

The transmission coefficient of the greenhouse covering material was estimated by calculating the ratio of PAR measured inside and outside the greenhouse using a quantum sensor (model LI-190R; LI-COR, Lincoln, NE, USA). The resulting transmission coefficient was 0.60.

### 2.6. Decision Tree-Based Classification of Greenhouse Microclimate

A categorical variable termed microclimate status was created to summarize the degree of suitability of internal greenhouse conditions relative to the optimal ranges defined for tomato. Daily microclimate status was modeled using Classification and Regression Trees (CART) [38], implemented with the rpart package in R.

Predictors consisted of the daily percentages of hours in optimal, suboptimal, and critical categories for T, RH, and VPD. The response was defined by a majority rule: if ≥2 of the 3 variables fell within the same category for more than 50% of the day, that category was assigned as the daily microclimate status.

Model complexity (cp) was tuned using rolling-origin cross-validation, which is particularly appropriate for time-ordered data [38]. The 1-SE rule was then applied before cost-complexity pruning.

Final performance was assessed on a temporal holdout set consisting of the last 30% of the time series. Evaluation metrics included confusion matrices, overall accuracy, balanced accuracy, macro F1-score, and Cohen’s κ, to account for both class imbalance and chance agreement [39].

Additionally, to gain a better understanding of the factors that lead to Critical microclimates, the number of hours for all the climatic ranges, detailed in Table 1, was calculated for each variable and sensor. Later, these values were converted to percentages for posterior analysis.

## 3. Results

### 3.1. Microclimatic Characteristics of a Low-Tech Greenhouse in Chaltura, Imbabura

The internal microclimatic conditions of the greenhouse were analyzed using descriptive statistics for temperature, relative humidity, and vapor pressure deficit, based on data collected by the four internal sensors throughout the entire production cycle. The results are summarized in Table 3.

The results highlight substantial spatial variability in the internal microclimate in the greenhouse, particularly for temperature and relative humidity. The differences detected among sensors indicate the presence of distinct microenvironments within the greenhouse, likely influenced by the greenhouse’s orientation, ventilation patterns, cultivation lines orientation and localized differences in the condition of the plastic covering. Additionally, the vent-to-ground surface ratio of the studied greenhouse was 10.46%, which is substantially below the minimum thresholds recommended for efficient natural ventilation in cold and humid environments (≥30%) and tropical conditions (>45%). This limited vent area, combined with the less than 2 m separation from the adjacent greenhouse, likely restricted lateral air exchange and contributed to the observed spatial heterogeneity in temperature, relative humidity, and VPD.

Such variability suggests that plants were exposed to unequal climatic conditions depending on their location. To assess whether these differences were statistically significant, a one-way analysis of variance (ANOVA) was performed for each variable (Table 4).

The results revealed highly significant differences (*p* < 0.001) among sensors for all three climatic variables, confirming the presence of pronounced spatial heterogeneity within the greenhouse. This heterogeneity may directly influence plant physiological development, as well as the spatial distribution of yield and fruit traits.

**Figure 3:**
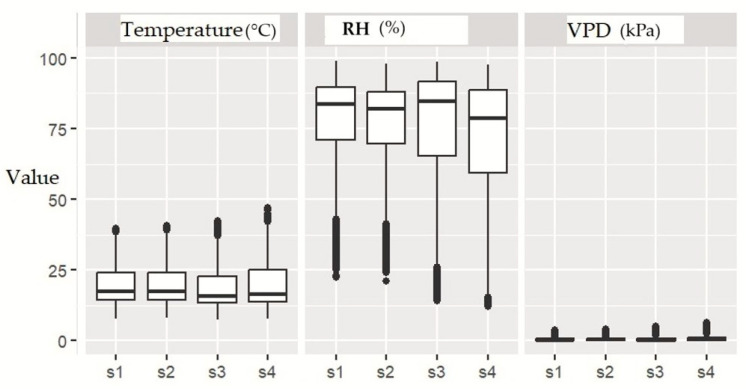
Comparative ANOVA Results for Temperature, Relative Humidity, and Vapor Pressure Deficit (VPD) Across Sensors.

Boxplots (Figure 3) illustrate the variability in temperature, relative humidity, and vapor pressure deficit recorded by each sensor, providing a clear visualization of distribution patterns and the occurrence of outliers.

### 3.2. External Climatic Conditions of the Low-Tech Greenhouse in Chaltura, Imbabura

The external climatic conditions recorded during the tomato production cycle are summarized in Table 5. These data provide essential context for interpreting the internal microclimate dynamics and their interaction with surrounding environmental conditions.

During the study period, average external temperatures ranged from 16 to 17 °C, with stable daily maximums of approximately 22–23 °C and dropping to 10 °C in August. The highest precipitation occurred in April (149.4 L m^−2^), coinciding with one of the early crop development stages, whereas September was the driest month (5.3 L m^−2^).

Average wind speed increased steadily, reaching 8 km h^−1^ in September, with prevailing easterly winds throughout the study period. Solar radiation remained consistently high across the entire production cycle.

According to the registered data, the average solar radiation in the research field was 189 W m^−2^ with no significant differences observed between phenological stages (*p* = 0.125).

### 3.3. Comparison Between Internal and External Conditions

A comparative analysis of internal and external conditions revealed significant differences, underscoring the greenhouse’s role in modifying the crop environment. The mean internal temperature was 19.15 °C, substantially higher than the external average of 16.34 °C and exhibited much greater variability (SD = 6.66 °C vs. 0.53 °C). The differences between internal and external temperature measurements were higher in the daytime (3.37 °C) than in the nighttime (1.44 °C). Differences were also recorded for phenological stages, finding the following average values for vegetative, reproductive, and harvest stages: 2.9, 2.07, and 2.45 °C, respectively.

The mean internal relative humidity was 76.47%, slightly higher than the external of 72.89%, but with a considerably larger standard deviation (16.29% vs. 7.10%). In addition, the relative humidity in the greenhouse was higher than the registered outside through 66.97% during daytime measurements. Contrarily, during nighttime measurements, an opposite effect was observed in 68.83% of the time. In addition, these differences in relative humidity were also affected by the phenological stages. As the crop advanced in development stages, higher was the proportion of time for which the relative humidity of the greenhouse was higher than that registered outside, showing the following percentages of differences for vegetative, reproductive, and harvest stages: 21.62%, 45.83%, and 66.11%, respectively.

**Figure 4:**
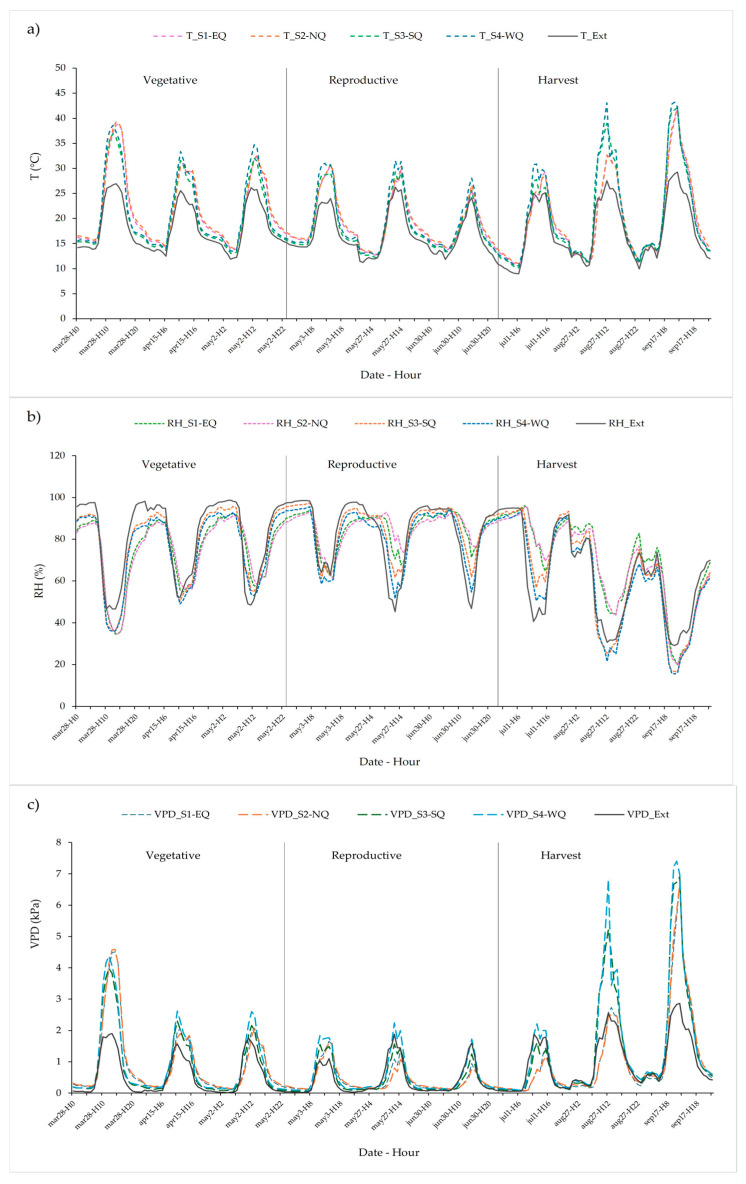
Temporal dynamics of internal and external microclimatic variables during representative days of each phenological stage. (**a**) Temperature, (**b**) Relative Humidity (RH), and (**c**) Vapor Pressure Deficit (VPD) recorded hourly by four internal sensors (dashed lines) and one external sensor (solid line) over three selected days per stage (vegetative, reproductive, and harvest). The plots illustrate diurnal and nocturnal patterns and highlight the coupling and decoupling between internal and external conditions.

The internal microclimate exhibited pronounced temporal and spatial variability throughout the tomato crop cycle. Figure 4 illustrates the hourly evolution of temperature, relative humidity (RH), and vapor pressure deficit (VPD) recorded by the four internal sensors (S1–S4), compared with external conditions. Clear diurnal and nocturnal patterns were observed, with daytime temperature peaks and nighttime humidity increases typical of passive greenhouses in highland regions. These dynamics were consistent across phenological stages but varied in magnitude among sensors, reflecting the spatial heterogeneity previously identified through ANOVA.

### 3.4. Microclimate Classification by Phenological Stage and Sensor Quadrant

To better understand the internal climatic dynamics of the greenhouse, the microclimate was classified into three categories (optimal, suboptimal, and critical), based on the integration of temperature, relative humidity, and vapor pressure deficit. This classification was performed using a decision tree approach, identifying the most influential thresholds and conditions for each category. In addition, independent analyses of T, RH, and VPD were conducted to capture variable-specific dynamics and their temporal variation across phenological stages (Appendix C). This dual approach provides both an integrated assessment of microclimate suitability and a detailed examination of the main environmental drivers affecting crop development.

The results of this classification for each sensor quadrant, phenological stage, and day–night times are summarized in Table 6.

The analysis summarizes the percentage distribution of optimal, suboptimal, and critical climatic conditions across phenological stages, day–night times, and sensor quadrants. When comparing the ranges formed by the minimum and maximum percentages of all sensors for each variable, it becomes evident that during the daytime, the crop was under optimal microclimatic conditions less than 50% of the time in all phenological stages, with values ranging from 15.5% to 48%. In contrast, the proportion of time under critical conditions ranged from 21.2% to 58%, resulting in an average optimum-to-critical ratio of 1.1. At night, the prevalence of optimal conditions decreased markedly, with values between 0.6% and 26.4%, and the optimum-to-critical ratio dropped to 0.2.

When analyzing the factors contributing to critical microclimates, it was observed that, on average across all phenological stages, the crop was exposed to critically low and high T for 20.6% and 4.7% of the time, respectively. Critical low T were more frequent at night (11.1%), whereas critical high T occurred mainly during the daytime (4%). Regarding RH, the crop experienced critically low and high values during 9.6% and 37.7% of the production cycle, respectively. Low RH was more common during the daytime (9.5%), while critically high RH dominated at night (30.2%). Similarly, VPD values were outside the optimal range for a large proportion of the cycle, with critically low and high values observed during 46.2% and 10.3% of the time, respectively. Low VPD occurred primarily at night (33.3%), whereas high VPD was more common during the daytime (10.3%).

When comparing phenological stages, it was evident that the reproductive phase was the most vulnerable, particularly during nighttime, when critical conditions exceeded 80% across all sensors. In contrast, the vegetative stage exhibited lower levels of critical exposure, especially during daytime, while the harvest stage presented a more balanced distribution of critical conditions between day and night. These patterns indicate that the reproductive stage coincided with less favorable microclimatic conditions that could potentially affect pollination and fruit set processes, while the harvest phase occurred under environments that may favor post-harvest quality deterioration or disease development.

To identify the dominant factor leading to critical microclimatic conditions, an independent analysis of temperature (T), relative humidity (RH), and vapor pressure deficit (VPD) was performed by phenological stage and time of day (Appendix C). The results showed that RH was the main determinant of critical conditions during nighttime, exceeding 80% in the vegetative and reproductive phases, whereas VPD exerted greater influence during daytime, particularly in the harvest stage, where critical conditions surpassed 60%. Temperature exhibited a lower relative contribution, although episodes of daytime thermal stress were observed in the vegetative phase. These findings confirm that control strategies should prioritize nighttime humidity regulation and daytime VPD management to minimize exposure to adverse microclimates.

### 3.5. Productive and Economic Indicators of Tomato

Harvesting began in the first week of July 2024 and was systematically carried out every Monday and Thursday until the end of the cycle on 19 September. A total of 24 commercial harvests were completed. Table 7 summarizes the cumulative yield and its distribution by quality category according to the local market.

The total yield reached 7.73 kg per plant, which is below the mean reported for the province of Imbabura. Nevertheless, 76.2% of the total production corresponded to marketable fruit in the first and second categories, while 23.8% was classified into categories destined for non-premium markets or processing.

The combined proportion of first- and second-grade fruit surpassed the 70% threshold typically considered acceptable for low-tech systems in the Andean region. This indicates that, despite the documented microclimatic heterogeneity, crop management practices maintained a high share of commercially viable fruit. However, the proportion of third- and fourth-grade fruit (23.8%) suggests room for improvement particularly in regulating RH and T during fruit set and development stages.

Table 8 presents the economic analysis of the tomato production cycle, detailing production costs including both direct and indirect costs, gross income, net profit, and the benefit–cost ratio.

The economic analysis revealed that pest and disease management represented a considerable share of production costs. Specifically, agrochemical inputs for pest control accounted for approximately 22% of total costs, while those for disease control represented 17%. These inputs were applied with a high frequency—often on a weekly basis—using a wide range of active ingredients for both insecticides and fungicides. This intensive management reflects the constant pressure of biotic factors within the greenhouse, likely exacerbated by the nocturnal excess humidity detected in the microclimatic analysis. Consequently, microclimate constraints not only affected physiological processes but also imposed significant economic implications, reducing the profitability margin of the production system.

## 4. Discussion

Protected cropping systems represent a technological framework with high transformative potential for small- and medium-scale horticultural production. This approach extends the growing season and improves yields, but its effectiveness depends heavily on the proper management of the internal microclimate [40,41]. In this study, the ANOVA results confirmed marked spatial heterogeneity in the greenhouse environment, with statistically significant differences in T, RH, and VPD among the installed sensors. These findings are consistent with previous research indicating that artisanal or low-tech greenhouses often exhibit uneven microclimatic distributions due to geometry, orientation, cover characteristics, management practices, and slope [11,13,42,43,44].

Regarding T, the differences observed between internal and external greenhouse conditions align with previous reports [45,46,47]. However, unlike the decreasing trend in internal T during advanced phenological stages reported by [45,48] attributed to greater canopy transpiration and shading our results did not show a clear reduction at harvest. This deviation may be explained by the grower’s pruning practices and by the severe drought that affected Ecuador in 2024, altering external climate conditions and disrupted electricity supply for prolonged periods, aggravating stress conditions [49,50,51].

For RH, our findings corroborate [45], which observed higher daytime RH inside greenhouses compared with outdoors and lower nighttime RH. Importantly, in our study the proportion of time with excessive RH increased as the crop advanced, reflecting insufficient air renewal. This trend coincides with results from [48], which demonstrated that phenological development strongly influences RH dynamics in protected environments.

In this sense, ventilation emerged as the most limiting factor. The vent-to-ground surface ratio of 10.46% in the studied greenhouse is substantially below the thresholds recommended for efficient air exchange. Tüzel et al. [52] suggested minimum ratios of 17–25% depending on the use of insect-proof nets, while [44] recommends values above 30% for cold and humid environments and more than 45% for warm and humid tropical conditions. Furthermore, the proximity of the adjacent greenhouse (<2 m) reduced lateral airflow, reinforcing the need to consider spacing and vent distribution in future designs to improve microclimatic uniformity. Similarly, refs. [43,53] demonstrated through CFD simulations that larger side and roof vent areas substantially improve airflow, homogenize internal climate, and reduce temperature gradients. The insufficient ventilation area likely contributed to the high daytime VPD and the persistence of critical RH values at night.

It is important to note that sensors were installed at a fixed height above the canopy throughout the crop cycle. As the canopy grew, the distance between sensors and the upper foliage decreased, which may have influenced airflow and localized humidity patterns. Previous studies have reported vertical microclimatic stratification in greenhouses, particularly affecting temperature and VPD during advanced phenological stages [12]. Future research should incorporate multi-level sensor placement to better capture these vertical variations and their impact on crop physiology and disease risk. Moreover, the orientation of the structure relative to prevailing winds strongly influences air exchange; winds perpendicular to the sidewalls maximize ventilation, whereas parallel winds limit renewal [54].

Radiation values recorded in this study are consistent with [55], which reported that inter-Andean valleys exhibit high and relatively stable global horizontal irradiance throughout the year. This relative constancy of external solar input suggests that the observed microclimatic variability inside the greenhouse was primarily shaped by structural and management factors rather than external variability.

The microclimate classification analysis revealed that the reproductive stage was the most vulnerable, particularly during nighttime, when critical conditions exceeded 80% across all sensors. In contrast, the vegetative stage exhibited lower levels of critical exposure, especially during daytime, while the harvest stage presented a more balanced distribution of critical conditions between day and night. These results indicate that reproductive processes such as pollination and fruit set are especially sensitive to adverse microclimates, while harvest conditions may exacerbate post-harvest quality losses and disease incidence.

From a physiological perspective, elevated RH and low VPD at night can impair pollen viability and fruit set. According to Harel et al. [56] and Archana et al. [57] tomato pollination is optimal when RH is maintained between 50 and 70%, whereas values above 90% increase susceptibility to thermal stress and reduce fruit set. In our case, the combination of high RH (>80%) and limited air renewal during the reproductive stage likely constrained reproductive efficiency.

Critical nighttime RH values also create favorable conditions for fungal pathogens. Ref. [58] demonstrated that nocturnal ventilation reduced condensation and *Botrytis cinerea* incidence in unheated tomato greenhouses, while [59] found that cultural practices such as wider plant spacing and polyethylene mulching suppressed humidity-related diseases in basil. Reference [60] further showed that spatial and vertical heterogeneity in protected environments generates microzones with dew formation, increasing foliar disease risk. These findings suggest that the high fungicide use observed in low-tech systems of the region may be directly related to persistent critical nighttime humidity.

Mitigation strategies in such contexts should focus on low-cost practices: improved nocturnal ventilation with insect-proof meshes, avoidance of late-afternoon irrigation, adoption of anti-drip plastic films, and regulated pruning and defoliation to improve air circulation. Additionally, biofungicides and biological control agents can help reduce pathogen pressure while decreasing dependence on chemical inputs.

Although the proportion of first- and second-grade fruits (76.2%) exceeded the minimum 70% threshold for commercial acceptance in the Andean region, the total yield (7.73 kg plant^−1^) was lower than averages reported for Imbabura greenhouses (12.07 kg m^−2^) and far from potential yields of 20 kg plant^−1^ under optimal conditions. The observed relationship between fruit classification and microclimatic conditions should be interpreted with caution. While temperature, relative humidity, and vapor pressure deficit (VPD) influence fruit development and quality traits, other agronomic factors such as soil fertility, irrigation, pruning, and fertilization also play a critical role. Therefore, our findings should be considered exploratory and context-specific; nonetheless, they align with previous studies showing that non-uniform microclimates can directly affect stem elongation, fruit growth, cluster weight, and overall yield [9,10,11,12,13,14,15,60]. Consequently, this analysis provides a preliminary foundation for future integrated studies that combine environmental, agronomic, and edaphic variables to better explain yield and quality variability under local greenhouse conditions.

The interactions documented in this study highlight the vulnerability of low-tech greenhouses to external forcing and internal design constraints. Optimizing the greenhouse microclimate requires integrated strategies combining structural design (orientation, vent distribution, roof height), passive measures (thermal screens, mulching, shading nets) [61,62,63], and simple technological tools such as IoT-based sensor networks for real-time monitoring [64,65]. These approaches can reduce the time crops remain under critical conditions and enable timely, data-driven decision-making by farmers.

Scaling-up of low-tech protected agriculture requires supportive public policies and targeted investment in technologies adapted to local realities. As emphasized by [41,61,66], coupling optimized microclimate management with cultural practices and affordable innovations can improve productivity while reducing dependence on chemical inputs, contributing to more sustainable and resilient protected horticulture systems in Andean regions [67,68].

Although tomato is the predominant greenhouse crop in Imbabura and Pietro F1 is among the most commonly used cultivars in the region, this study was conducted in a single low-tech greenhouse (Block 2) under specific management and agroecological conditions. Therefore, caution should be exercised when generalizing the findings to other greenhouse structures or production systems within Csb climate zones of Ecuador. Future research involving multiple sites and cultivars is needed to validate and expand upon these results.

While this study covered a single crop cycle, the continuous monitoring of temperature and relative humidity at five-minute intervals across all phenological stages provides a robust dataset for microclimatic characterization. Furthermore, this research constitutes the first scientific documentation of internal climate behavior in low-tech greenhouses in the Imbabura province, offering a foundational reference for future longitudinal and multi-location studies.

Even though the link between excessive humidity and increased disease risk is well documented, this study provides empirical evidence of how persistent critical nighttime RH levels in low-tech greenhouses directly correlate with high pesticide application frequency and elevated production costs. By integrating microclimatic monitoring with economic analysis, our findings highlight the tangible impact of microclimatic stress on both plant health and farm profitability. This context-specific quantification is particularly relevant for Andean regions, where technical limitations and climatic variability exacerbate vulnerability in protected cropping systems.

Overall, the findings of this study reflect broader challenges faced by low-tech greenhouse systems in the Andean region of Ecuador, where artisanal structures with limited environmental control are predominant. The documented microclimatic heterogeneity and its agronomic and economic implications underscore the need for targeted interventions. Looking forward, the development of locally adapted climate-control strategies, integration of affordable sensor technologies, and capacity-building for growers are essential to enhance the sustainability and resilience of protected horticulture in Csb climate zones. These priorities should guide future research and policy efforts aimed at strengthening the sector.

## 5. Conclusions

Low-tech protected agriculture offers a strategic pathway to enhance food security, increase productivity, and promote sustainability in regions with challenging agroclimatic conditions. However, its effectiveness is significantly constrained by critical limitations, particularly in microclimate management, financial resources, and technical capacity.

This study demonstrated that spatial heterogeneity in greenhouse microclimate can substantially limit tomato yield, highlighting the importance of continuous environmental monitoring and the implementation of climate-control strategies tailored to local conditions. The results revealed that excessive nighttime humidity not only increased the risk of physiological and pathological stress but also led to a greater reliance on fungicides and insecticides, which together accounted for nearly 40% of total production costs.

These findings underscore that microclimate regulation has both agronomic and economic implications. Improving internal climate control is essential to reduce input dependency, enhance profitability, and advance toward more sustainable protected horticulture systems in the Andean region. Future efforts should focus on integrating passive ventilation improvements, affordable sensor technologies, and context-specific management practices to strengthen the resilience and efficiency of low-tech greenhouse agriculture.

## 6. Recommendation

To improve microclimatic homogeneity and reduce excessive nighttime humidity in low-tech greenhouses, it is recommended to increase the total ventilation surface area, particularly through the expansion of lateral and roof openings. This structural adjustment can enhance air exchange, decrease relative humidity during critical periods, and reduce the incidence of fungal diseases, thereby lowering the frequency of pesticide applications and improving overall system sustainability.

Additionally, the adoption of simple yet effective agronomic practices should be prioritized. These include strategic shading to mitigate daytime overheating, passive heating techniques to buffer nocturnal cooling, and regulated pruning and defoliation to improve internal airflow. The use of anti-drip polyethylene films and the avoidance of late-afternoon irrigation can further help minimize condensation and disease pressure.

For small- and medium-scale farming systems, integrating affordable sensor networks for real-time climate monitoring can support data-driven decision-making and optimize resource use. These interventions, when adapted to local agroecological conditions, can significantly enhance the resilience, productivity, and profitability of protected horticulture in Andean regions.

## Figures and Tables

**Table 1:** Optimal, Suboptimal and Critical Climatic Ranges by Phenological Stage for Tomato.

Phenological Stage	Time of Day	Climatic Variable	Optimal Range	Suboptimal Range	Critical Range
Vegetative (0–50 DAT *)	Day	T (°C)	20–28	18–19.9 o 28.1–34	<18 o >34
		RH (%)	55–75	50–54 o 76–85	<50 o >85
		** VPD (kPa)	0.5–1.1	0.4–0.49 o 1.2–2.0	<0.4 o >2.0
	Night	T (°C)	15–19	12–14.9 o 19.1–20	<12 o >20
		RH (%)	50–75	45–49 o 76–80	<45 o >80
		** VPD (kPa)	0.5–0.9	0.3–0.49 o 1.0–1.5	<0.3 o >1.5
Reproductive (51–109 DAT *)	Day	T (°C)	19–26	17–18.9 o 26.1–34	<17 o >34
		RH (%)	50–80	45–49 o 81–89	<45 o >89
		** VPD (kPa)	0.5–1.2	0.4–0.49 o 1.3–2.0	<0.4 o >2.0
	Night	T (°C)	15–19	13–14.9 o 19.1–20	<13 o >20
		RH (%)	50–75	45–49 o 76–85	<45 o >85
		** VPD (kPa)	0.5–0.9	0.3–0.49 o 1.0–1.5	<0.3 o >1.5
Harvest (110–190 DAT *)	Day	Temp. (°C)	19–24	17–18.9 o 24.1–34	<17 o >34
		RH (%)	50–80	45–49 o 81–89	<45 o >89
		** VPD (kPa)	0.5–1.2	0.4–0.49 o 1.21–2.0	<0.4 o >2.0
	Night	Temp. (°C)	15–19	13–14.9 o 19.1–20	<13 o >20
		RH (%)	50–75	45–49 o 76–85	<45 o >85
		** VPD (kPa)	0.5–0.9	0.3–0.49 o 1.0–1.5	<0.3 o >1.5

* DAT is days after transplant; ** VPD is expressed in absolute values.

**Table 2:** Duration of Phenological Stages in Tomato.

Phenological Stage	Start Date	End Date	Duration (Days)
Vegetative (0–50 DAT)	14 March 2024 (* Day 74)	2 May 2024 (* Day 123)	50
Reproductive (51–109 DAT)	3 May 2024 (* Day 124)	30 June 2024 (* Day 182)	59
Harvest (110–190 DAT)	1 July 2024 (* Day 183)	19 September 2024 (* Day 263)	81
Total			190

* DAY refers to the Julian day number in the 2024 calendar year (e.g., DAY 1 = 1 January; DAY 74 = 14 March).

**Table 3:** Descriptive Statistics of Temperature (T), Relative Humidity (RH), and Vapor Pressure Deficit (VPD) Recorded by Internal Sensors.

Phenological Stage	Variable	Sensor	Mean	Min Absolute	Max Absolute	Mean of Min	Mean of Max
Vegetative	T (°C)	S1-EQ	20.3	9.8	39.8	13.7	34.1
		S2-NQ	20.7	10.1	40.8	13.9	34.8
		S3-SQ	19.2	9.1	38.5	12.8	32.2
		S4-WQ	20.2	9.3	39.6	13.2	34.4
	RH (%)	S1-EQ	75.0	27.0	95.0	43.2	89.8
		S2-NQ	74.7	25.5	95.0	43.8	88.8
		S3-SQ	78.1	31.0	97.0	44.9	94.1
		S4-WQ	75.4	29.5	96.1	42.6	92.7
	VPD (kPa)	S1-EQ	0.6	0.1	3.8	0.1	2.4
		S2-NQ	0.6	0.08	4.1	0.2	2.5
		S3-SQ	0.6	0.04	3.5	0.08	2.1
		S4-WQ	0.6	0.06	3.7	0.1	2.4
Reproductive	T (°C)	S1-EQ	18.7	11.1	33.8	13.2	29.4
		S2-NQ	19.1	11.2	35.0	13.5	30.2
		S3-SQ	18.1	10.7	33.3	12.7	28.9
		S4-WQ	19.7	10.9	37.2	12.9	32.5
	RH (%)	S1-EQ	84.4	53.0	98.5	63.5	94.8
		S2-NQ	85.3	55.5	98.0	68.5	94.8
		S3-SQ	84.0	44.5	98.5	57.0	95.3
		S4-WQ	78.3	35.2	97.5	48.9	93.5
	VPD (kPa)	S1-EQ	0.3	0.03	1.7	0.07	1.2
		S2-NQ	0.3	0.03	1.6	0.08	1.0
		S3-SQ	0.4	0.02	2.1	0.06	1.4
		S4-WQ	0.5	0.04	2.8	0.09	1.9
Harvest	T (°C)	S1-EQ	18.9	7.9	38.0	10.9	29.2
		S2-NQ	18.8	8.2	40.3	11.3	29.3
		S3-SQ	17.7	7.4	42.3	10.4	31.2
		S4-WQ	18.5	7.8	47.2	10.8	36.0
	RH (%)	S1-EQ	75.8	22.5	99.0	55.9	93.4
		S2-NQ	71.4	21.0	98.0	54.8	91.3
		S3-SQ	71.9	14.0	98.2	43.1	90.7
		S4-WQ	65.8	12.1	94.4	31.6	85.9
	VPD (kPa)	S1-EQ	0.6	0.01	3.8	0.08	1.5
		S2-NQ	0.6	0.03	4.2	0.12	1.5
		S3-SQ	0.7	0.03	5.0	0.1	2.1
		S4-WQ	0.8	0.08	6.5	0.2	3.2

S1-EQ = Eastern Quadrant; S2-NQ = Northern Quadrant; S3-SQ = Southern Quadrant; S4-WQ = Western Quadrant.

**Table 4:** ANOVA Results of Microclimatic Variables Across Sensors.

	Df	Sum Sq	Mean Sq	F Value	Pr (>F)
**One-way ANOVA for temperature**					
Sensor	3	35,981	11,994	269.3	<2 × 10^−16^ ***
Residuals	146,136	6,508,741	45		
**One-way ANOVA for relative humidity**					
Sensor	3	578,139	192,713	717.8	<2 × 10^−16^ ***
Residuals	146,136	39,233,980	268		
**One-way ANOVA for vapor pressure deficit**					
Sensor	3	673	224.40	479.9	<2 × 10^−16^ ***
Residuals	146,136	65,862	0.45		

Significance codes: 0, “***”.

**Table 5:** External Climatic Parameters During the Tomato Production Cycle in Chaltura, Imbabura.

Parameter	Criteria	Vegetative (0–50 DAT)	Reproductive (51–109 DAT)	Harvest (110–190 DAT)
T (°C)	Mean	16.54	16.27	16.23
	Min absolute	9	10	7
	Max absolute	26	27	26
	Mean of Min	12.6	12.42	10.80
	Mean of Max	22.72	22.34	22.98
RH (%)	Mean	77.04	79.59	64.18
	Min absolute	27	29.5	18.5
	Max absolute	99.5	99	95.5
	Mean of Min	42.7	47.1	33.9
	Mean of Max	92.3	96	87.4
Precipitation (L m^−2^)	Total by phenological stage	162.5	99.3	46.3
Wind	Mean_speed (km h^−1^)	4.68	4.80	7.78
	Predominant direction	E	E	E
Solar radiation (W m^−2^)	Mean	198.2	188.8	180
	Mean_Max	1168.5	1150	1145.6

Source: Local Climatological Data. Davis Instruments, WeatherLink Network.

**Table 6:** Microclimate Classification (%) by Phenological Stage, Day–Night Times, and Sensor Quadrant for Tomato Crop Cultivated in a Low-Tech Greenhouse in Chaltura, Imbabura.

Sensor S1-EQ					
Phenological Stage	Time of Day	Optimal (%)	Suboptimal (%)	Critical (%)	Total (%)
Vegetative	Day	33.5	22.8	43.7	100
Vegetative	Night	11.6	28.9	59.5	100
Reproductive	Day	45.8	29.2	25	100
Reproductive	Night	1.2	14.8	84	100
Harvest	Day	28.8	27.4	43.8	100
Harvest	Night	15.8	27.2	57.0	100
Average	Day	36.0	26.5	37.5	100
Average	Night	9.5	23.6	66.8	100
Sensor S2-NQ					
Phenological Stage	Time of Day	Optimal (%)	Suboptimal (%)	Critical (%)	Total (%)
Vegetative	Day	30.4	28.6	41.0	100
Vegetative	Night	15.6	33.5	50.9	100
Reproductive	Day	40.1	28.4	31.5	100
Reproductive	Night	1.0	18.9	80.1	100
Harvest	Day	30.6	25.1	44.3	100
Harvest	Night	18.8	35.8	45.4	100
Average	Day	33.7	27.4	38.9	100
Average	Night	11.8	29.4	58.8	100
Sensor S3-SQ					
Phenological Stage	Time of Day	Optimal (%)	Suboptimal (%)	Critical (%)	Total (%)
Vegetative	Day	48.0	21.4	30.6	100
Vegetative	Night	1.7	6.8	91.5	100
Reproductive	Day	43.1	35.6	21.3	100
Reproductive	Night	0.6	5.2	94.2	100
Harvest	Day	28.0	29.4	42.6	100
Harvest	Night	18.0	25.4	56.6	100
Average	Day	39.7	28.8	31.5	100
Average	Night	6.8	12.5	80.8	100
Sensor S4-WQ					
Phenological Stage	Time of Day	Optimal (%)	Suboptimal (%)	Critical (%)	Total (%)
Vegetative	Day	44.4	21.9	33.7	100
Vegetative	Night	2.6	13.4	84.0	100
Reproductive	Day	34.7	44.1	21.2	100
Reproductive	Night	3.0	11.9	85.1	100
Harvest	Day	15.5	26.5	58	100
Harvest	Night	26.4	37.9	35.7	100
Average	Day	31.5	30.8	37.6	100
Average	Night	10.7	21.1	68.3	100

**Table 7:** Crop Yield by Quality Category (kg ha^−1^).

Category	Weight (kg ha^−1^)	Share (%)
First	37,507.7	42.6%
Second	29,591.8	33.6%
Third	16,295.6	18.5%
Fourth	4607.3	5.3%
Total	88,002.5	100%

**Table 8:** Economic indicators of tomato cultivated in a low-tech greenhouse in Chaltura, Imbabura.

Variable	Value (USD ha^−1^)
Total cost	30,800.9
Gross Income	44,001.3
Gross profit	13,200.4
Benefit–cost ratio (B/C)	1.4

## Data Availability

The data supporting the findings of this study are available from the corresponding author upon reasonable request.

## Appendix A

**Table A1:** Technical specifications of CS300-TH sensors—Milesight, China.

Feature	Specification
Transmission technology	LoRaWAN^®^
Compatible frequencies	CN470, IN865, RU864, EU868, US915, AU915, KR920, AS923
Transmission power	16 dBm (868 MHz), 20 dBm (915 MHz), 19 dBm (470 MHz)
Sensitivity	−137 dBm @ 300 bps
Operating modes	OTAA/ABP (Clase A)
Temperature range	−30 °C a + 70 °C
Temperature accuracy	±0.3 °C (0–70 °C), ±0.6 °C (−30–0 °C)
Temperature resolution	0.1 °C
Relative humidity range	0–100% HR
Relative humidity accuracy	±3% (10–90% HR), ±5% (<10% o >90% HR)
Relative humidity resolution	0.5% HR

## Appendix B

**Table A2:** Technical specifications of the Davis Instruments Vantage Vue^®^ station—Davis Instruments, USA.

Feature	Specification
Model	Vantage Vue^®^
Update frequency	Up to every 2.5 s
Wireless transmission range	Up to 300 m with spread spectrum technology
Data storage capacity	Up to 180 days (depending on logging interval)
Sensor compatibility	Integration with over 80 additional sensor types
Platform and connectivity	WeatherLink Live (Wi-Fi/Ethernet)
Data access	Real-time and historical via app and WeatherLink website
Virtual integration	Compatible with Amazon Alexa and Google Assistant

## Appendix C

**Table A3:** Microclimate Classification (%) by Phenological Stage, Time of Day, and Independent Variables of Sensor S1-EQ.

Sensor S1-EQ Temperature (T)					
Phenological Stage	Time of Day	Optimal (%)	Suboptimal (%)	Critical (%)	Total (%)
Vegetative	Day	38.7	34.7	26.5	100
Vegetative	Night	56.9	37.6	5.5	100
Reproductive	Day	43.9	36.0	20.1	100
Reproductive	Night	53.3	36.3	10.4	100
Harvest	Day	26.7	43.5	29.8	100
Harvest	Night	26.7	31.2	42.1	100
Average	Day	36.5	38.1	25.5	100
Average	Night	45.6	35.0	19.3	100
Sensor S1-EQ Relative Humidity (RH)					
Phenological Stage	Time of Day	Optimal (%)	Suboptimal (%)	Critical (%)	Total (%)
Vegetative	Day	41.8	23.5	34.7	100
Vegetative	Night	5.8	9.7	84.6	100
Reproductive	Day	56.6	20.4	23.0	100
Reproductive	Night	1.1	13.8	85.1	100
Harvest	Day	40.2	21.9	37.9	100
Harvest	Night	25.9	37.5	36.6	100
Average	Day	46.2	21.9	31.9	100
Average	Night	10.9	20.3	68.7	100
Sensor S1-EQ Vapor pressure deficit (VPD)					
Phenological Stage	Time of Day	Optimal (%)	Suboptimal (%)	Critical (%)	Total (%)
Vegetative	Day	21.1	34.7	44.1	100
Vegetative	Night	8.1	23.7	68.2	100
Reproductive	Day	43.9	23.2	32.9	100
Reproductive	Night	2.0	12.1	85.8	100
Harvest	Day	30.4	19.9	49.8	100
Harvest	Night	15.4	27.2	57.4	100
Average	Day	31.8	25.9	42.3	100
Average	Night	8.5	21.0	70.5	100

**Table A4:** Microclimate Classification (%) by Phenological Stage, Time of Day, and Independent Variables of Sensor S2-NQ.

Sensor S2-NQ Temperature (T)					
Phenological Stage	Time of Day	Optimal (%)	Suboptimal (%)	Critical (%)	Total (%)
Vegetative	Day	34.5	40.1	25.4	100
Vegetative	Night	57.6	33.9	8.5	100
Reproductive	Day	41.6	38.8	19.6	100
Reproductive	Night	56.9	33.2	9.9	100
Harvest	Day	24.6	45.1	30.2	100
Harvest	Night	30.1	30.5	39.4	100
Average	Day	33.6	41.4	25.1	100
Average	Night	48.2	32.5	19.3	100
Sensor S2-NQ Relative Humidity (RH)					
Phenological Stage	Time of Day	Optimal (%)	Suboptimal (%)	Critical (%)	Total (%)
Vegetative	Day	43.9	23.9	32.2	100
Vegetative	Night	9.2	13.4	77.4	100
Reproductive	Day	42.3	27.2	30.4	100
Reproductive	Night	0.9	17.6	81.4	100
Harvest	Day	42.4	19.4	38.1	100
Harvest	Night	33.8	37.8	28.4	100
Average	Day	42.9	23.5	33.6	100
Average	Night	14.6	22.9	62.4	100
Sensor S2-NQ Vapor pressure deficit (VPD)					
Phenological Stage	Time of Day	Optimal (%)	Suboptimal (%)	Critical (%)	Total (%)
Vegetative	Day	16.7	27.9	55.4	100
Vegetative	Night	11.5	30.4	58.1	100
Reproductive	Day	43.6	17.0	39.4	100
Reproductive	Night	2.7	15.9	81.4	100
Harvest	Day	30.6	20.0	49.4	100
Harvest	Night	18.8	34.3	46.9	100
Average	Day	30.3	21.6	48.1	100
Average	Night	11.0	26.9	62.1	100

**Table A5:** Microclimate Classification (%) by Phenological Stage, Time of Day, and Independent Variables of Sensor S3-SQ.

Sensor S3-SQ Temperature (T)					
Phenological Stage	Time of Day	Optimal (%)	Suboptimal (%)	Critical (%)	Total (%)
Vegetative	Day	42.0	33.3	24.6	100
Vegetative	Night	45.4	49.8	4.8	100
Reproductive	Day	42.0	39.3	18.7	100
Reproductive	Night	40.1	42.9	17.0	100
Harvest	Day	25.6	44.8	29.6	100
Harvest	Night	22.5	27.9	49.6	100
Average	Day	36.6	39.1	24.3	100
Average	Night	36.0	40.2	23.8	100
Sensor S3-SQ Relative Humidity (RH)					
Phenological Stage	Time of Day	Optimal (%)	Suboptimal (%)	Critical (%)	Total (%)
Vegetative	Day	38.7	26.5	34.7	100
Vegetative	Night	3.2	2.3	94.5	100
Reproductive	Day	67.2	13.8	19.0	100
Reproductive	Night	0.6	4.7	94.7	100
Harvest	Day	47.6	15.1	37.2	100
Harvest	Night	31.0	32.4	36.6	100
Average	Day	51.2	18.5	30.3	100
Average	Night	11.6	13.2	75.2	100
Sensor S3-SQ Vapor pressure deficit (VPD)					
Phenological Stage	Time of Day	Optimal (%)	Suboptimal (%)	Critical (%)	Total (%)
Vegetative	Day	18.8	37.6	43.7	100
Vegetative	Night	2.8	5.5	91.7	100
Reproductive	Day	37.4	32.3	30.3	100
Reproductive	Night	0.5	3.9	95.6	100
Harvest	Day	24.9	28.1	47.0	100
Harvest	Night	19.0	26.4	54.6	100
Average	Day	27.0	32.7	40.3	100
Average	Night	7.4	12.0	80.6	100

**Table A6:** Microclimate Classification (%) by Phenological Stage, Time of Day, and Independent Variables of Sensor S4-WQ.

Sensor S4-WQ Temperature (T)					
Phenological Stage	Time of Day	Optimal (%)	Suboptimal (%)	Critical (%)	Total (%)
Vegetative	Day	38.0	33.8	28.2	100
Vegetative	Night	53.9	42.9	3.2	100
Reproductive	Day	35.1	46.5	18.4	100
Reproductive	Night	47.2	40.7	12.1	100
Harvest	Day	16.5	46.0	37.5	100
Harvest	Night	26.1	32.8	41.1	100
Average	Day	29.9	42.1	28.0	100
Average	Night	42.4	38.8	18.8	100
Sensor S4-WQ Relative Humidity (RH)					
Phenological Stage	Time of Day	Optimal (%)	Suboptimal (%)	Critical (%)	Total (%)
Vegetative	Day	35.4	31.0	33.6	100
Vegetative	Night	3.9	4.8	91.2	100
Reproductive	Day	66.9	18.9	14.2	100
Reproductive	Night	2.8	13.4	83.8	100
Harvest	Day	29.6	16.1	54.3	100
Harvest	Night	48.2	36.0	15.8	100
Average	Day	44.0	22.0	34.0	100
Average	Night	18.3	18.1	63.6	100
Sensor S4-WQ Vapor pressure deficit (VPD)					
Phenological Stage	Time of Day	Optimal (%)	Suboptimal (%)	Critical (%)	Total (%)
Vegetative	Day	17.4	35.4	47.2	100
Vegetative	Night	4.1	11.8	84.1	100
Reproductive	Day	29.2	32.5	38.3	100
Reproductive	Night	2.0	10.1	87.9	100
Harvest	Day	14.2	23.9	61.9	100
Harvest	Night	27.2	37.8	35.0	100
Average	Day	20.3	30.6	49.1	100
Average	Night	11.1	19.9	69.0	100
